# Supplementary material for: Dynamic transcriptomic profiles of zebrafish gills in response to zinc supplementation
Source: BMC Genomics. 2010 Oct 11;11:553. doi: 10.1186/1471-2164-11-553 (PMC3091702; doi:10.1186/1471-2164-11-553)
Supplement: Additional file 2 — Interactive Direct Interaction Network representing the molecular interactions between zinc, copper, iron, calcium and proteins encoded by transcripts changed by zinc supplementation. Mini web-site containing index.html and hyperlinked pages in subdirectory describing a Direct Interaction Network automatically generated based on curated interactions contained within the proprietary PathwayArchitect database. Ovals represent proteins and the circles symbolize metal ions. Objects are coloured by their abundance in zebrafish at the time-point they were significantly different from the control is a scale from -4 fold (dark green) to +4 fold (dark red). Where significant differences were found at more than one time-point, the colour overlay shows expression at the first instance. Dark blue squares denote 'binding', and light blue squares 'expression'; green squares stand for 'regulation', green diamonds for 'metabolism', and green circles for 'promoter binding'. Arrow heads indicate directionality of the interaction where annotated. All nodes and edges can be further interrogated by selecting the relative area of the image. [file 1471-2164-11-553-S2.zip › PathwayArchitect Zn xs DIN/88711.html]

# PROTEIN: PDC

|  |  |
| --- | --- |
| Name | PDC |
| Type | PROTEIN |
| Description | phosducin |
| Note | This gene encodes a phosphoprotein, which is located in the outer and inner segments of the rod cells in the retina. This protein may participate in the regulation of visual phototransduction or in the integration of photoreceptor metabolism. It modulates the phototransduction cascade by interacting with the beta and gamma subunits of the retinal G-protein transducin. This gene is a potential candidate gene for retinitis pigmentosa and Usher syndrome type II. Alternatively spliced transcript variants encoding different isoforms have been identified. |
| Alias | Phototransducing protein 33 kDa (phosducin) |
|  | phosducin-like orphan protein |
|  | phosducin-like protein |
|  | 33kDA phototransducing protein |
|  | PhLP |
|  | PHD |
|  | G beta gamma binding protein |
|  | rod photoreceptor 1 |
|  | 33 kDa phototransducing protein |
|  | Rpr1 |
|  | MEKA protein |
|  | MEKA |
|  | PhLOP |
|  | Phototransducing protein, 33 kDa (phosducin) |
|  | Pdc |
|  | 33DPTP |
|  | Rpr-1 |
|  | RPR-1 |
|  | Rod photoreceptor 1 |
|  | PDC |


---

|  |  |
| --- | --- |
| GO Component | cytosol |
|  | soluble fraction |
|  | cytoplasm |


---

|  |  |
| --- | --- |
| GO ID | GO:0007186 |
|  | GO:0009586 |
|  | GO:0007600 |
|  | GO:0005489 |
|  | GO:0006118 |
|  | GO:0007601 |
|  | GO:0005625 |
|  | GO:0005737 |
|  | GO:0004859 |
|  | GO:0008277 |
|  | GO:0007602 |
|  | GO:0005095 |
|  | GO:0005829 |


---

|  |  |
| --- | --- |
| MIM | MIM:171490 |


---

|  |  |
| --- | --- |
| Connectivity | 270 |


---

|  |  |
| --- | --- |
| Entrez ID | 20028 |
|  | 5132 |
|  | 25343 |


---

|  |  |
| --- | --- |
| Agilent ID | A\_44\_P290488 |
|  | A\_43\_P11596 |
|  | A\_51\_P357914 |
|  | A\_14\_P201150 |
|  | A\_14\_P126927 |
|  | A\_53\_P164819 |
|  | A\_23\_P23090 |
|  | A\_53\_P143350 |


---

|  |  |
| --- | --- |
| Cellular Localization | Cytosol |
|  | Cytoplasm |
|  | Cell |


---

|  |  |
| --- | --- |
| Pathway | Zn xs inventory |
|  | Zn xs DIN |


---

|  |  |
| --- | --- |
| GO Process | phototransduction |
|  | G-protein coupled receptor protein signaling pathway |
|  | regulation of G-protein coupled receptor protein signaling pathway |
|  | electron transport |
|  | visual perception |
|  | rhodopsin mediated phototransduction |
|  | sensory perception |


---

|  |  |
| --- | --- |
| UniGene | Hs.550 |
|  | Mm.47709 |
|  | Rn.9739 |


---

|  |  |
| --- | --- |
| Affymetrix Probeset ID | 1369139\_at |
|  | 1449179\_at |
|  | 162267\_r\_at |
|  | 211496\_s\_at |
|  | 34603\_at |
|  | 94139\_at |
|  | g177186\_3p\_a\_at |
|  | L08075\_at |
|  | M33478\_at |
|  | M33528\_at |
|  | 100637\_at |


---

|  |  |
| --- | --- |
| GO Function | phospholipase inhibitor activity |
|  | GTPase inhibitor activity |
|  | electron transporter activity |


---

|  |  |
| --- | --- |
| Nucleotide | M38058 |
|  | BC093839 |
|  | NM\_024458 |
|  | L20420 |
|  | BC037176 |
|  | L08075 |
|  | BQ636049 |
|  | M33530 |
|  | M60738 |
|  | M38059 |
|  | M33478 |
|  | NM\_012872 |
|  | AL596220 |
|  | NM\_022576 |
|  | M60720 |
|  | AK020798 |
|  | M33528 |
|  | AF172075 |
|  | NM\_002597 |
|  | BC012507 |
|  | BC021920 |
|  | AF076464 |


---

|  |  |
| --- | --- |
| Protein | AAH93839 |
|  | AAH12507 |
|  | AAA40603 |
|  | AAA40604 |
|  | NP\_077778 |
|  | AAD43142 |
|  | P20942 |
|  | AAK13559 |
|  | Q9QW08 |
|  | P20941 |
|  | NP\_002588 |
|  | NP\_037004 |
|  | NP\_072098 |
|  | CAI13122 |
|  | AAA36210 |
|  | AAH21920 |
|  | AAA35486 |
|  | AAA41841 |


---

|  |  |
| --- | --- |
| Organism | Mammal |


---

|  |  |
| --- | --- |
| Location | chromosome 1, 1q25.2 (Homo sapiens) |
|  | chromosome 1, 1 80.0 cM, 1 G1 (Mus musculus) |
|  | 1 80.0 cM (Mus musculus) |
|  | chromosome 13, 13q21 (Rattus norvegicus) |


---

|  |  |
| --- | --- |
